# Developing an exercise intervention to minimise hip bone mineral density loss following traumatic lower limb amputation: a Delphi study

**DOI:** 10.1136/bjsports-2024-108721

**Published:** 2024-09-03

**Authors:** Fearghal P Behan, Anthony M J Bull, Belinda R Beck, Katherine Brooke-Wavell, Ralph Müller, Laurence Vico, Hanna Isaksson, Nicholas C Harvey, Arjan Buis, Kate Sherman, Gemma Jefferson, Daniel J Cleather, Alison McGregor, Alexander N Bennett

**Affiliations:** 1 Imperial College London, London, UK; 2 Trinity College Dublin, Dublin, Ireland; 3 Department of Bioengineering, Imperial College London, London, UK; 4 Griffith University, Brisbane, Queensland, Australia; 5 Department of Human Sciences, Loughborough University, Loughborough, UK; 6 Institute of Biomechanics, ETH Zurich, Zurich, Switzerland; 7 Inserm U1059 SAINBIOSE, Université Jean Monnet Saint-Etienne, Saint-Priest-en-Jarez, France; 8 Department of Biomedical Engineering, Lund University, Lund, Sweden; 9 MRC Lifecourse Epidemiology Unit, University of Southampton, Southampton, UK; 10 University of Strathclyde, Glasgow, UK; 11 Dorset Orthopaedic, Cheshire, UK; 12 UK Sports Institute, Manchester, UK; 13 School of Sport, Health and Applied Science, St. Mary's University, Twickenham, London, UK; 14 Surgery and Cancer / Human Performance Group, Imperial College London, London, UK; 15 Academic Department of Military Rehabilitation, Defence Medical Rehabilitation Centre, Epsom, UK

**Keywords:** Exercise, Exercise Therapy, Bone density, Bone, Lower limb

## Abstract

**ABSTRACT:**

**Objective:**

To elicit expert opinion and gain consensus on specific exercise intervention parameters to minimise hip bone mineral density (BMD) loss following traumatic lower limb amputation.

**Methods:**

In three Delphi rounds, statements were presented to a panel of 13 experts from six countries. Experts were identified through publications or clinical expertise. Round 1 involved participants rating their agreement with 22 exercise prescription statements regarding BMD loss post amputation using a 5-point Likert scale. Agreement was deemed as 3–4 on the scale (agree/strongly agree). Statements of <50% agreement were excluded. Round 2 repeated remaining statements alongside round 1 feedback. Round 3 allowed reflection on round 2 responses considering group findings and the chance to change or maintain the resp onse. Round 3 statements reaching ≥70% agreement were defined as consensus.

**Results:**

All 13 experts completed rounds 1, 2 and 3 (100% completion). Round 1 excluded 12 statements and added 1 statement (11 statements for rounds 2–3). Round 3 reached consensus on nine statements to guide future exercise interventions. Experts agreed that exercise interventions should be performed at least 2 days per week for a minimum of 6 months, including at least three different resistance exercises at an intensity of 8–12 repetitions. Interventions should include weight-bearing and multiplanar exercises, involve high-impact activities and be supervised initially.

**Conclusion:**

This expert Delphi process achieved consensus on nine items related to exercise prescription to minimise hip BMD loss following traumatic lower limb amputation. These recommendations should be tested in future interventional trials.

**WHAT IS ALREADY KNOWN ON THIS TOPIC**

- Individuals with traumatic lower limb amputations have reduced bone mineral density (BMD) of the hip on their amputated side. Exercise interventions have been shown to improve BMD in other populations, but this has not been evaluated in a population with lower limb amputation.

**WHAT THIS STUDY ADDS**

- A team of international experts achieved a consensus on nine statements regarding the appropriate parameters of an exercise intervention to minimise hip BMD loss in those with traumatic lower limb amputation. These parameters will be used within a subsequent interventional trial to assess their feasibility, safety and efficacy within a population with traumatic limb loss.

**HOW THIS STUDY MIGHT AFFECT RESEARCH, PRACTICE OR POLICY**

- The present findings have been driven by best existing empirical evidence alongside world leading expert opinion and may act as a current guide for practice until future well-controlled experiments are completed in this population.

## Introduction

 Traumatic lower limb amputations impose a large health burden and lead to significant disability.^1^,^3^ Globally, the incidence and prevalence of traumatic amputations are increasing,^1 4^ partially due to an increase in road traffic accidents.^5^ It is known that lower limb amputation results in reduced hip bone mineral density (BMD) of men who have had a traumatic amputation (greater in above than below knee amputations),^6 7^ and this is deemed to result from a localised unloading osteopenia.^7^ Reduced hip BMD can lead to osteoporosis, heightening the risk of hip fractures, both associated with significant societal, personal and economic costs.^8^ This risk is compounded in those with limb loss as they are already at a greater risk of falling.^9^ Furthermore, this could have a disproportionate effect in individuals with an amputation as it could compromise the short- and long-term use of prosthetic devices leading to functional and mobility limitations.

Exercise interventions to improve BMD have been successful in other populations: space flight,^10^ postmenopausal women^11^ and in individuals recovering from anorexia.^12^ Expert consensus statements^13^ alongside systematic reviews with meta-analyses,^11^ have recommended specific exercise loading protocols to optimise BMD parameters. However, exercise loading to reduce hip BMD loss following lower limb amputation has not been well documented. Exercise in populations with intact limbs may not be suitable for those with an amputation as the transmission of force is mediated non-physiologically through a socket or in some cases, an osseointegration implant. Biomechanical transmission of the load to the proximal femur requires careful consideration for individuals with lower limb loss.

Despite the lack of empirical evidence on exercise interventions in this population, anecdotally clinicians have been attempting to minimise BMD loss following an amputation. Furthermore, the relatively young age of many of those following a traumatic amputation^5 14^ may result in the need for, but may also facilitate, more rigorous exercise interventions than previous interventions in older populations.^15^ Consequently, there is a pressing need to establish biomechanically driven loading parameters and subsequently determine the safety, feasibility and efficacy of these exercise interventions in a rigorous and systematic fashion in those with lower limb loss.^16^

However, prior to implementing any intervention it would be prudent to leverage clinical expertise, scientific evidence from clinical trials and biomechanical knowledge in related fields, to assist in developing successful interventions and develop a foundation of evidence-based practice. Using the Delphi technique has proven valuable as this process can generate knowledge that can provide insights into interventional parameters and their potential effectiveness prior to implementation, also facilitating clinical expertise which cannot be extracted from systematic reviews.^17^ The objective of this study was to elicit expert opinion and gain a consensus to define a viable exercise intervention to minimise hip BMD loss following a traumatic lower limb amputation.

## Methods

### Study design

We selected the Delphi method for this study as it allowed us to achieve an expert consensus asynchronously and remotely.^18^ Additionally, the Delphi technique allows individuals worldwide to participate and use their expertise, facilitates participants to remain anonymous from one another, and prevents social conformity to a dominant view (the bandwagon effect).^19 20^ Delphi processes have been implemented successfully in a variety of different clinical settings.^21^,^23^ Therefore, prior to implementing an interventional study, current expert knowledge was leveraged to ensure an optimal protocol with the available current evidence base and expertise through the Delphi process. This allowed specific questions to be answered regarding the exact parameters to include in any subsequent interventional study: how frequently the intervention should be completed, what intensity should the intervention be prescribed at, what the duration of the intervention should be and what type of interventional exercises should be used?^24 25^ Guidelines on conducting and reporting Delphi studies have been adhered to in this process.^19^

### Steering committee

A multidisciplinary steering group was formed to develop and conduct this Delphi process consisting of relevant disciplines (physiotherapy, exercise science, rheumatology, rehabilitation, sports and exercise medicine, bioengineering, musculoskeletal biomechanics) and research expertise (quantitative methods, interventional trial development and implementation, longitudinal trial management, computational musculoskeletal and biomechanics modelling).^16^ Agreement was reached regarding inclusion and exclusion criteria of the expert committee, structure and content of statements, and analysis procedures, using previous Delphi studies and guidelines for guidance.^1921^,^23 26^

### Generation of the statement list

The statements were split into four sections according to a well-established exercise science framework, the F.I.T.T. (frequency, intensity, time, type) principle of exercise prescription.^24 25^ The statements were developed to provide the expert panel with summarised conclusions from best available empirical outcomes related to the exposure and outcome of interest. The parameters included in the statements were generated through the findings of systematic reviews/meta-analyses,^1115 27^,^29^ interventional clinical trials^1012 30^,^38^ and clinical guidelines^13 39^ using exercise as a stimulus for BMD change.

### Selection of international experts

Participants were selected through purposeful sampling. They were deemed suitable if they were seen as experts in a relevant field by the steering committee. Participants were deemed as experts if they were:

1. Author of two or more English language peer-reviewed publications related to the domain (ie, improving BMD) or constructs (ie, exercise prescription)^22 23^ and/or
2. Have 5+ years of clinical experience of prescribing exercise interventions in those with limb loss. Participants were excluded if they did not have sufficient academic or clinical domain specific expertise or if they did not consent to participation. To form a representative international expert panel, we included a diverse range of professions, research and clinical practice disciplines (physiotherapy, rheumatology, rehabilitation, sports and exercise medicine, engineering, biology, prosthetics, strength and conditioning), countries (aiming for a spread of at least five countries) and backgrounds.^16^ We aimed for a panel of 10–15 experts, inviting 20 experts initially. As there are no explicit recommendations on Delphi sample size we aimed for this sample as it is similar to previous Delphi studies^23 40^ and as within fields with limited experts, such as the current field, strict inclusion criteria allows for effective and reliable utilisation of a moderate number of experts.^41^ We aimed for at least 40% of the experts to be either practicing clinically or have a clinical background, and at least 40% from a scientific/engineering background.^16^

### Patient and public involvement

Three individuals with lower limb amputations formed a study patient and public involvement group and were involved in the early conception of study design and two individuals gave further detailed input based on their experience and preferences on the structure of the statements and the potential implementation of the intervention, with statements adapted accordingly.

### Equity, diversity and inclusion

This study was gender balanced, with seven females and seven males involved, including junior investigators. The Delphi participants are from a mixture of ethnicities and nationalities. This work is targeted towards those with lower limb loss, a largely underrepresented population within sports medicine research, thus this work explicitly aims to improve inclusion and diversity within this field.

### Anonymity

The iterative nature of a Delphi technique means that participants are anonymous to each other, but not to the researcher, deemed quasi-anonymity.^20 42 43^ On completion of the process, participants were offered the choice to receive personal acknowledgement and give input to the future publication for their involvement.^20^

### Delphi procedure

Each stage of the Delphi study involved piloting the survey to a group of four to six postdoctoral researchers familiar with the disciplines to ensure comprehensibility of statements, effective survey set-up and accurate interpretation and analysis of data.^16 23^ Construction, distribution and data collection was conducted remotely as an e-Delphi using Microsoft Forms (Microsoft, Redmond, WA, USA). All participants were sent a pre-Delphi invitation email alongside a letter explaining the background and aims of the study. Participants completed a demographic questionnaire and a consent form agreeing to complete the three rounds of the Delphi process.^44^

### Round 1

Round 1 consisted of participants being asked to rate their level of agreement with 22 statements using a 5-point Likert scale (0=strongly disagree, 1=disagree, 2=neither agree nor disagree, 3=agree, 4=strongly agree). Agreement was deemed as 3 or 4 on the Likert scale. The statements were split into four established domains related to exercise prescription: frequency (2 statements), intensity (2 statements), time (6 statements) and type (12 statements). The first round contained an open-ended question at the end of each section for feedback from participants on improvements/modifications required for round 2.^23^ The answers to the statements were analysed for percentage agreement, with those statements receiving less than 50% agreement among experts being excluded from progressing to round 2^23^ to reduce the list of items least likely to achieve consensus^45^ and to reduce the number of statements in the final two rounds. This reduced number after round 1 was aimed towards reducing the risk of fatigue and drop-out in the Delphi expert participant panel. Each round was open for 2 weeks, with a week between for analysis. If there was no response in week 1, participants were reminded at the beginning of week 2, and if there had still been no response participants received a further personalised reminder on the final day to minimise attrition.

### Round 2

Round 2 consisted of the same statements as round 1 with any alterations to the statements (terminology, clarity, additional statements) based on round 1 feedback and the exclusion of any statement that failed to reach at least a 50% consensus from round 1.^45^ Other than the exclusion of questions from round 1 and additions based off participant feedback, no explicit feedback from group results of round 1 were given in round 2. Participants were again asked to rate their level of agreement using the same scale and categorisation of statements. One final open-ended question in round 2 allowed for additional comments/inputs to be added prior to the final third round. Round 2 statements were analysed for percentage agreement with ≥70% (3 or 4 on the Likert scale) deemed to achieve provisional consensus pending round 3 input.^23^

### Round 3

Each participant was presented with their individual response and asked to reflect on their round 2 responses alongside statistical representation (percentage agreement) of group opinion for each statement to inform their responses to round 3 and whether they wished to alter any of their round 2 responses accordingly.^19 22 23^ This allowed participants to realise disparities between them and the rest of the experts, to reconsider the evidence or to reflect on and re-evaluate their answer for each statement based on the group statistics.^22 23^ Statements reaching predefined criteria (ie, ≥70% of the expert group scoring their responses as 3 or 4 on the Likert scale)^19 45^ were deemed to reach final consensus and were to be implemented (where possible) in the exercise intervention.

## Results

We invited 20 people to participate in the Delphi process, 13 experts agreed to participate, 5 did not response and 2 felt they did not have sufficient expertise to participate. In all three rounds, all 13 experts participated fully (100% completion). The demographics of the experts are included in table 1.

**Table 1:** Expert panel demographics (mean (SD))

Age	Years of experience	Gender	Professional area of expertise	Countries
52 (7)	25 (8)	Seven females, six males	Four clinical (two physiotherapists, two consultant rheumatologists),Nine academic (five bone mechanobiologists, one bioengineer, one physiotherapist, one strength and conditioning coach, one prosthetist)	Australia, England, France, Scotland, Sweden, Switzerland

All authors of this manuscript except the first author were participants in the Delphi process, two authors were also members of the initial steering group. For a detailed breakdown of the statements and results of each round see table 2.

**Table 2:** Delphi statements and percentage agreement per round

Statement	Round 1 agreement (%)	Round 2 agreement (%)	Round 3 agreement (%)	Consensus reached
**Frequency**				
1. A minimum of 2 days per week of an exercise intervention is required to increase bone mineral density (BMD)	85, continued to round 2	85	92	**Yes**
2. Only one day per week of an exercise intervention is sufficient to increase BMD	8, excluded from round 2	Excluded	Excluded	No
**Intensity**				
3. Resistance exercises in this intervention should be completed at an intensity of 8–12 repetition maximum to increase BMD (ie, a load which causes repetition failure ≤12 repetitions)	59, continued to round 2	83	83	**Yes**
4. Resistance exercises in this intervention should be completed at an intensity of five repetition maximum to increase BMD	36, excluded from round 2	Excluded	Excluded	No
**Time**				
5. The exercise intervention should last at least 6 months to increase BMD	85, continued to round 2	100	100	**Yes**
6. The exercise intervention would only require 12 weeks or less to increase BMD	8, excluded from round 2	Excluded	Excluded	No
7. Each interventional session should contain at least three different resistance exercises to increase BMD	83, continued to round 2	69	77	**Yes**
8. Only 1–2 resistance exercises are required per interventional session to increase BMD	9, excluded from round 2	Excluded	Excluded	No
9. A minimum of three sets will be required per exercise to increase BMD	36, excluded from round 2	Excluded	Excluded	No
10. Only 1–2 sets per exercise will be sufficient to increase BMD	9, excluded from round 2	Excluded	Excluded	No
**Type**				
11. Resistance training should be included in this exercise intervention to increase BMD	100, continued to round 2	100	100	**Yes**
12. Resistance training **alone** should be sufficient in this intervention to increase BMD.	18, excluded from round 2	Excluded	Excluded	No
13. Resistance training within this intervention should include weight bearing exercises to increase BMD (eg, squats, deadlifts, etc)	100, continued to round 2	92	100	**Yes**
14. Resistance training within this intervention should **only** include weight bearing exercises to increase BMD (eg, squats, deadlifts, etc)	10, excluded from round 2	Excluded	Excluded	No
15. Resistance training within this intervention should include non-weight bearing exercises with high muscle forces to increase BMD (eg, resisted hip abduction/adduction)	55, continued to round 2	33	27	No
16. The exercise programme should be multi-planar (sagittal, frontal, transverse)	Not included in round 1	75	92	**Yes**
17. Resistance training within this intervention should **only** include non-weight bearing exercises with high muscle forces to increase BMD (eg, resisted hip abduction/adduction)	8, excluded from round 2	Excluded	Excluded	No
18. Resistance training within this intervention should involve **both** weight bearing exercises and non-weight bearing exercises to increase BMD	77, continued to round 2	50	42	No
19. High impact exercises (eg, jumping, hopping, skipping) should be added alongside resistance training in this intervention to increase BMD	92, continued to round 2	85	92	**Yes**
20. High impact exercises **alone** (without resistance training) should be sufficient in this intervention to increase BMD	38, excluded from round 2	Excluded	Excluded	No
21. Aerobic exercise should also be included in this programme alongside other interventions to increase BMD (resistance training, high impact exercise)	45, excluded from round 2	Excluded	Excluded	No
22. Whole-body vibration should be included within this programme during the exercise intervention to increase BMD	18, excluded from round 2	Excluded	Excluded	No
23. All interventional sessions should be initially supervised by a suitably trained individual	54, continued to round 2	77	83	**Yes**

BMDbone mineral density

### Round 1 outcomes

Round 1 consisted of 22 initial statements. 12 statements received less than 50% consensus and were therefore excluded (50% of total round 1 statements). After analysis of the expert feedback from round 1, an extra statement was added for round 2.

### Round 2 outcomes

Round 2 consisted of 11 initial statements (10 initial statements plus an additional added statement from round 1 feedback, table 2). Nine of these statements achieved provisional consensus (82% of total round 2 statements), that is, ≥70% of the expert group scoring their responses as ≥3 on the Likert scale.

### Round 3 outcomes

**Figure 1:**
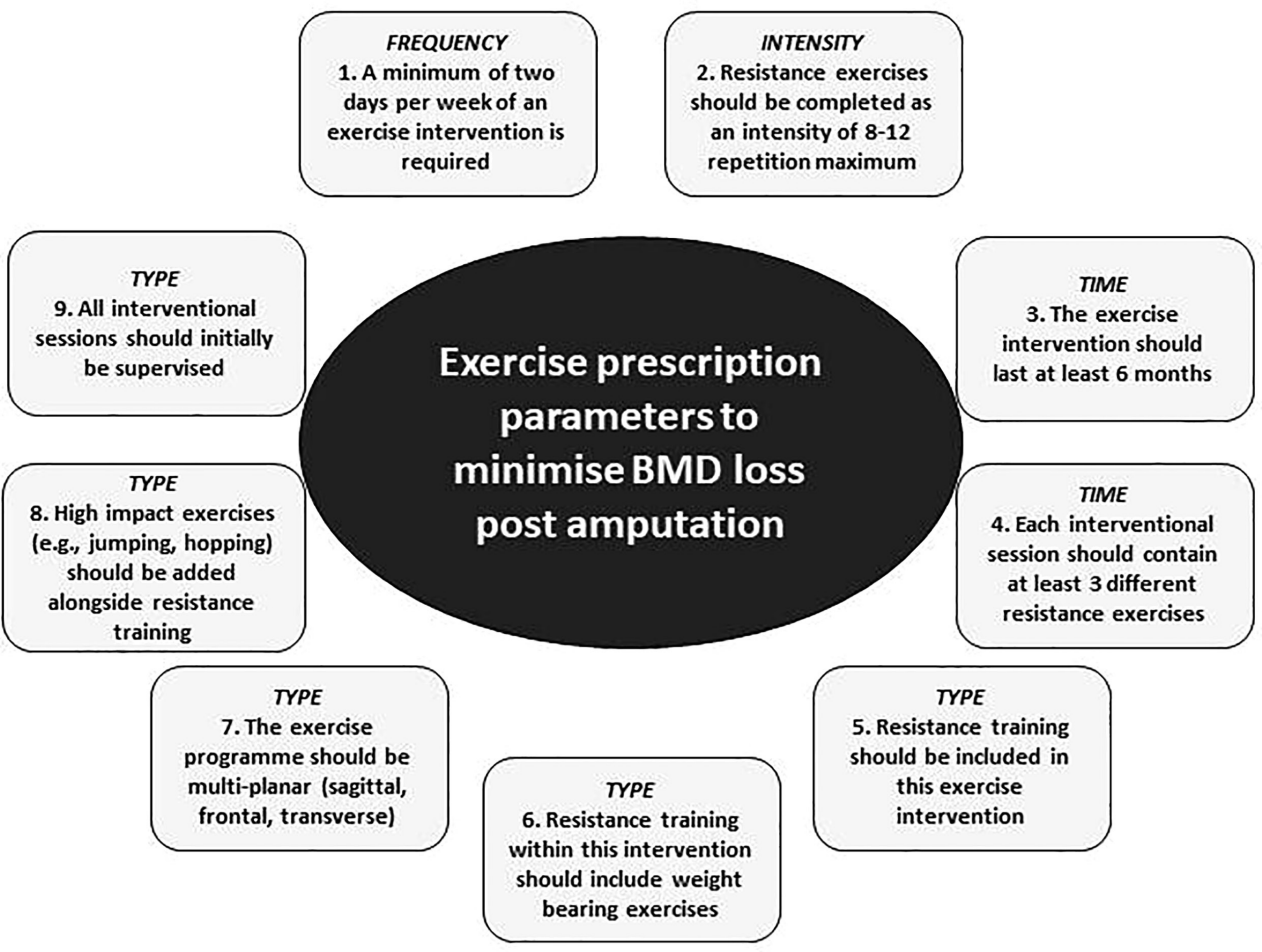
Final statements to reach consensus after round 3. BMD, bone mineral density.

Following participants being presented with group results, percentage agreement changed for 7 of the 11 statements between round 2 and round 3. However, none of these changes were large enough to reduce any agreement on a statement from ≥70% to below, or below 70% to above. Therefore, nine of the statements were deemed to reach consensus (39% of total statements (23) reaching consensus, 61% excluded) regarding exercise prescription parameters to increase BMD following lower limb loss (figure 1). One statement from the frequency domain, one statement from the intensity domain, two statements from the time domain and five statements from the type domain ultimately achieved consensus.

The experts agreed that the exercise intervention should be performed at least 2 days per week for a minimum of 6 months, including at least three different resistance exercises per session at an intensity of 8–12 repetitions. The intervention should include resistance training with weight bearing and multiplanar exercises, incorporate high-impact activities and should initially be supervised.

## Discussion

The present paper elicited expert opinion and obtained a consensus on nine statements regarding the appropriate parameters of an exercise intervention to minimise hip BMD loss in those with lower limb amputation. These parameters will be used within a subsequent interventional trial to assess their feasibility, safety and efficacy within a population with traumatic limb loss. The results of these studies have the potential to provide clarity on exercise prescription based on expert opinion in amputated individuals and if the intervention is proven successful, may stabilise or improve hip BMD, and potentially reduce lifelong fracture risk and associated functional limitation and psychosocial morbidity within this population.

The experts achieved consensus on one statement from the frequency domain, one statement from the intensity domain, two statements from the time domain and five statements from the type domain, based on the F.I.T.T. principle of exercise prescription.^24 25^ Frequency was agreed on by experts as a minimum of 2 days per week (statement 1), in accordance with the vast majority of literature.^28 31 32 34^ The inclusion of a variety of different resistance training exercises (statement 5: resistance training should be included, statement 4: each intervention should contain at least three different resistance exercises) including weight bearing resistance training (statement 6) is largely consistent with the interventions used in interventional studies that have improved BMD.^28 31^ Recent interventions^31 35^ and recommendations^13^ include weight bearing exercise despite previous recommendations for non-weight bearing exercises prior to more contemporary trials.^29^

Experts also agreed that high impact exercises (eg, jumping, hopping) should be included alongside resistance training (statement 8). This is consistent with recent successful trials,^31^ however, trials of high impact exercise alone have also proven successful for improving BMD.^35^ Although multi-planar exercises were agreed on to be included in the intervention (statement 7), and have also been included in previous interventions to provide variably distributed loads to the bone (bending, twisting, compression),^35^ previous interventions have also focused on predominantly sagittal plane exercises with successful results for femoral neck BMD.^31^

Experts agreed on an intensity of 8–12 repetitions during resistance training (statement 2). This is a repetition range that has been commonly used and recommended in previous studies to improve BMD.^13 27 36^ However, a lower prescription of 5 repetitions has also proven beneficial,^31^ as well as repetition ranges of 6–8 repetitions.^37 38^ It would appear that a large range of repetitions may be possible to increase BMD once a sufficient osteogenic stimulus is achieved. Ensuring initial supervision for the sessions reached consensus (statement 9), in accordance with previous trials^3335^,^38^; this facilitates the option for remote supervision to reduce potential logistical strain of the intervention.^46^ A duration of >6 months reached consensus (statement 3), consistent with interventional trials that have increased BMD.^31 35^

Initially, a short-term feasibility study may be warranted due to the novelty of investigating many elements of the intervention, largely extrapolated from populations with bilateral intact lower limbs, in a population with lower limb amputation. The addition of a specific exercise loading programme to increase hip BMD in a population who have suffered an amputation seems pertinent considering recent evidence that neither walking^7^ nor sporting activity^47^ appear enough to prevent bone loss in the hip following amputation. The target of this work will be individuals who have suffered traumatic amputations, as those with cancer-related or dysvascular amputations^48^ may require more individualised, adapted exercise regimes due to comorbidities.^16 49^

Exercise in populations with intact limbs may not be suitable for individuals with limb loss as the interface between the force applied and the femoral neck is mediated non-physiologically through a socket (or osseointegration implant). Exercise adaptations and progressive bone loss in those with an amputated lower limb are likely to be different from other populations due to the offloading of the residual limb by the prosthetic socket design, reducing loading in the femoral neck.^6^ Biomechanical differences between those with above knee amputations and below knee amputations will also require careful consideration prior to any large-scale exercise implementation.^50^ If it is assumed that clinicians have already been attempting to increase BMD in those with lower limb amputations, this is occurring with minimal guidance from empirical data due to a dearth of experimental studies on the effects of mechanical loading on bone density changes following amputation.^47^ Consequently, there is a pressing need to establish appropriate loading parameters in this population. We have obtained a set of exercise prescription guidelines in the current study from a group of world experts driven by experimental evidence in related populations and clinical experience and expertise. Many different training approaches could be employed to meet the criteria defined by the agreed statements, the next steps will be to develop an intervention that adheres to these guidelines and determine the safety, feasibility and efficacy of this intervention in a controlled and systematic fashion in individuals with lower limb loss. The additional issues of exercise adherence and patient preference will also need to be considered in subsequent trial design. By implementing this recommended exercise intervention to focus on loading the femoral neck, direct recommendations could be disseminated on the success of exercise interventions prior to any potential other intervention, such as socket alteration.

Using the Delphi procedure provides benefits where there is limited direct clinical knowledge of the effectiveness of an intervention as it can generate knowledge that may provide guidance prior to the large logistical task of implementation.^17^ However, without direct interventional data on the effect of exercise on BMD in a population with lower limb loss, the Delphi process cannot create new evidence for this, but only infer recommendations in a population of individuals with limb loss based on empirical data from exercise interventions on BMD in other populations,^10 13 27 30 31^ alongside current scientific knowledge of this population.^67 9 51^,^53^ Therefore, clinical recommendations on exercise interventions to increase BMD in those with limb loss will still require future rigorous, controlled, experimental trial data due to the inherent limitations of expert consensus methodologies (including the assumption that a multitude of associated perspectives is superior to an individual perspective, lack of cause-effect findings, the possibility to question the composition of the expert group, etc).^17^ Our panel lacked representation from low-to-middle income countries, something we plan to remedy in our follow-up interventional work. Our stringent selection criteria alongside 25% of invitees not responding and 10% not feeling they had adequate knowledge, limited our sample size and potential breadth of expertise. Our statements may be at risk of a ceiling effect as three of our statements that reached consensus asked for a minimum value (statement 1: ‘A minimum of 2 days per week …’; statement 3: ‘… should last at least 6 months …’; and statement 4: ‘… at least three different resistance exercises …’), not facilitating the experts to give a potential higher value for the exercise prescription that they may have wished to. Thus, the recommendation may be seen as a minimum dose.

Alongside implementing the recommendations from our Delphi process in interventional trials, a series of technical studies (computational and laboratory) would be additive to fill further mechanical knowledge gaps regarding proximal force propagation to the residual limb and the femoral neck in those with limb loss. Previously Delphi procedures have been used for exercise prescription in clinical conditions^54^ including those with osteoporosis,^39^ but none have been performed to define a biomechanically driven loading intervention to increase BMD in those with lower limb loss, adding novelty to the literature. The present findings have been driven by best existing empirical evidence alongside world leading expert opinion and may act as a current guide for practice until future well-controlled experiments are completed in this population.

## Conclusion

This is the first time an expert Delphi process has been aimed towards minimising the effect of localised unloading osteopenia in people with traumatic lower limb amputation. The process achieved consensus on nine items related to exercise prescription to improve BMD following traumatic lower limb loss. The exercise intervention should be performed at least 2 days per week for a minimum of 6 months, including at least three different resistance exercises per session at an intensity of 8–12 repetitions. The intervention should include resistance training with weight-bearing and multiplanar exercises, incorporate high-impact activities and should initially be supervised. The results of this Delphi will be used to design a feasibility interventional trial with the best available scientific knowledge available to optimise safety and maximise potential positive clinical outcomes in those with lower limb loss.

## Data Availability

Data are available upon reasonable request.
